# Mechanochemical Formation of Racemic Praziquantel Hemihydrate with Improved Biopharmaceutical Properties

**DOI:** 10.3390/pharmaceutics12030289

**Published:** 2020-03-23

**Authors:** Debora Zanolla, Dritan Hasa, Mihails Arhangelskis, Gabriela Schneider-Rauber, Michele R. Chierotti, Jennifer Keiser, Dario Voinovich, William Jones, Beatrice Perissutti

**Affiliations:** 1 Department of Chemical and Pharmaceutical Sciences, University of Trieste, P.le Europa 1, 34127 Trieste, Italy; debora.zanolla@gmail.com (D.Z.); dhasa@units.it (D.H.); 2 Faculty of Chemistry, University of Warsaw, 1 Pasteura Street, 02-093 Warsaw, Poland; m.arhangelskis@uw.edu.pl; 3 Department of Chemistry, University of Cambridge, Lensfield Road, CB2-1EW Cambridge, UK; gabisrauber@gmail.com (G.S.-R.); wj10@cam.ac.uk (W.J.); 4 Department of Chemistry and NIS Centre, University of Torino, V. Giuria 7, 10125 Torino, Italy; michele.chierotti@unito.it; 5 Swiss Tropical and Public Health Institute, Socinstrasse 57, P.O. Box, CH-4002 Basel; Switzerland; jennifer.keiser@swisstph.ch; 6 Universität Basel, Petersplatz 1, P.O. Box, CH-4001 Basel, Switzerland

**Keywords:** praziquantel, hemihydrate, mechanochemistry, neat grinding, liquid-assisted grinding, racemic compound, polymorphism, crystal structure solution

## Abstract

Praziquantel (PZQ) is the first-line drug used against schistosomiasis, one of the most common parasitic diseases in the world. A series of crystalline structures including two new polymorphs of the pure drug and a series of cocrystals of PZQ have been discovered and deposited in the Cambridge Structural Database (CSD). This work adds to the list of multicomponent forms of PZQ a relevant example of a racemic hemihydrate (PZQ-HH), obtainable from commercial PZQ (polymorphic Form A) through mechanochemistry. Noteworthy, the formation of the new hemihydrate strongly depends on the initial polymorphic form of PZQ and on the experimental conditions used. The new PZQ-HH has been fully characterized by means of HPLC, Differential Scanning Calorimetry (DSC), Thermogravimetric Analysis (TGA), Hot-Stage Microscopy (SEM), Powder X-Ray Diffraction (PXRD), Scanning Electron Microscopy (SEM), FT-IR, polarimetry, solid-state NMR (SS-NMR), solubility and intrinsic dissolution rate (IDR), and in vitro tests on *Schistosoma mansoni* adults. The crystal structure was solved from the powder X-ray diffraction pattern and validated by periodic-DFT calculations. The new bioactive hemihydrate was physically stable for three months and showed peculiar biopharmaceutical features including enhanced solubility and a double intrinsic dissolution rate in water in comparison to the commercially available PZQ Form A.

## 1. Introduction

In the last few years, significant attention has been dedicated to the improvement of therapies for neglected tropical diseases, including schistosomiasis. Indeed, around 240 million people are currently affected by schistosomiasis, as reported by the World Health Organization [1], and it has been placed third (after malaria and intestinal helminthiases) in the rank of the main tropical diseases [2]. Schistosomiasis is caused by the infection by blood flukes of the genus *Schistosoma* and, depending on the species, chronic worsening can lead to bladder cancer, kidney failure, liver fibrosis, intestinal and urinary diseases, etc. [3]. Currently, praziquantel (PZQ) represents the drug of choice against all species of *Schistosoma*, with a therapeutic regimen of 20 mg/kg three times a day at intervals of 4 to 6 h or as a single dose of 40 mg/kg (depending on the parasite and whether used for individual patient management or mass drug administration) [3,4]. Such a high dosage is mainly due to the poor water solubility and bioavailability and extensive first-pass metabolism [5]. Very minor and transient side effects [6] and low toxicity in animals [7] are reported for PZQ. The low toxicity together with the possibility of administration in pregnant women and children under 24 months [8] and the lack of alternative treatment options are some of the main reasons why PZQ is the main choice for the treatment of schistosomiasis. 

**Figure 1:**
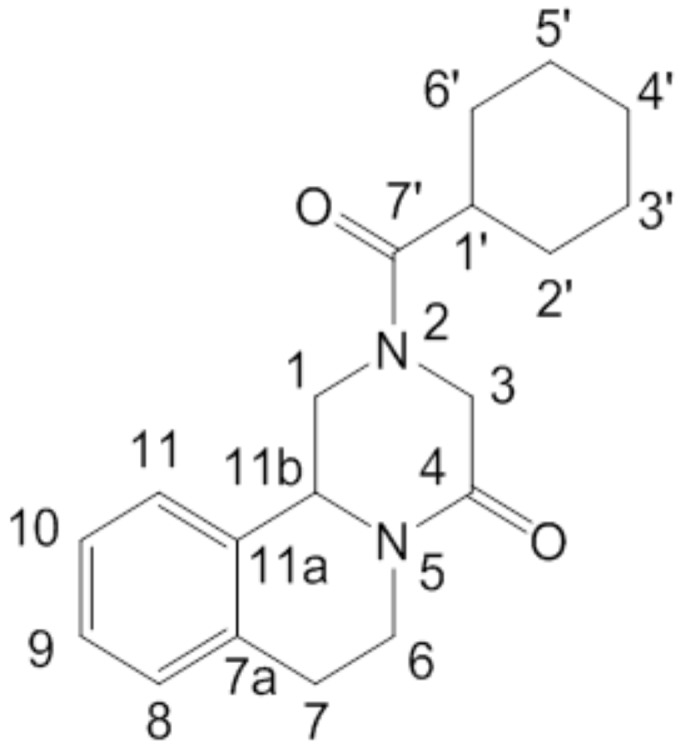
Chemical structure of PZQ ((11bRS)-2-(Cyclohexylcarbonyl)-1,2,3,6,7,11b-hexahydro-4-H-pyrazino[2,1-a]isoquinolin-4-one) with atom numbering.

PZQ (Figure 1) is currently used in the racemic form, even though the pharmacological activity is mainly driven by the (R)-enantiomer [9,10,11,12], which also has a much higher bioavailability [13]. Moreover, (S)-PZQ (the inactive enantiomer) has been recognized as being responsible for the bitter taste [14] and worsened side effects [15,16]. 

The commercial form of PZQ is a racemic anhydrate structure, indexed as TELCEU [17] in the Cambridge Structural Database (CSD) [18]. The crystal structures of the pure enantiomers are also available in CSD as hemihydrates (CSD codes SIGBUG, SIGBUG01) [14,15]. In the context of improving the biopharmaceutical properties of commercial PZQ, recently, two additional anhydrate polymorphic forms (Form B and Form C) were reported by our group produced through neat grinding of commercial PZQ (Form A) for about 4 h in a vibrational mill. The two new polymorphs are also available in CSD (indexed as TELCEU01 and GOYZOM, respectively). Both PZQ Form B and Form C preserve the activity against *Schistosoma* and have an improved water solubility [19,20]. Very recently, other authors have proposed a further solid form, not indexed in the CSD, reported as a pink powder; however, the performed characterizations neither allowed to solve the structure nor to validate it [21]. Moreover, our group has also reported the possibility of obtaining PZQ in a highly amorphous state, physically stable for several months, by a mechanochemical treatment in the presence of different polymeric excipients [22]. Such solid dispersions, however, presented a diminished drug recovery, which was found to be dependent on the polymer used [23,24]. Conversely, when PZQ is processed alone by neat grinding maintains a conspicuous residual crystallinity and does not degrade [19,20].

Despite the absence of a hydrogen bond donor group in the molecular structure (Figure 1), PZQ shows an interesting solid-state behavior, forming a variety of multicomponent systems. Indeed, Espinosa-Lara et al. reported a series of PZQ cocrystals with different dicarboxylic acids (i.e., oxalic, malonic, succinic, maleic, fumaric, glutaric, adipic and pimelic acids) obtained via liquid-assisted grinding in a vibrational mill, using acetone or acetonitrile as the added liquids for facilitating mechanochemical synthesis. The new solid forms were fully characterized, including single-crystal X-ray diffraction analysis and a full list of hydrogen bonding interactions, motifs and short contacts [17].

Here, we present a new hemihydrate of racemic PZQ, obtained through mechanochemistry. The study of hydrates is of great importance in the pharmaceutical field since several physico-chemical and biopharmaceutical properties can be affected by the insertion of water molecules in the crystal lattice [25]. Moreover, water is often present during several stages of drug manufacturing, thus hydrate formation is a very common, yet poorly understood phenomenon. It has been indeed estimated that one-third of the pharmaceutical molecules could exist as hydrates [26]. A possible explanation for the large number of hydrated forms known (representing about 11% of all structures recorded in CSD [27]) can rely on the fact that water molecules contain both donor and acceptor sites and can, therefore, create links both with itself and/or with other compounds, thus enhancing the possibility of additional intermolecular interactions. There have been some studies on the prediction of hydrate formation using computational methods, however, we are far from a prediction-only scenario [28]. Similar to other solvates, the most common situation in hydrates (about 50%) is one additional molecule for each one of the host molecules, while only 8% of the classes of hydrate are hemihydrates [28]. In a hydrate, the water molecules occupy fixed positions in the crystal lattice, either filling structural voids or being an integral part of the structure. This also implies a different moisture sorption/desorption behavior. As a consequence, hydrates are commonly classified into non-stoichiometric hydrates (frequently hosting water molecules in structural voids, subjected to reversible water uptake/release without significant changes in the crystal structure) and stoichiometric hydrates (leading to a different structure or an amorphous state after the escape of water molecules) [29,30]. Based on their structure, the hydrate nomenclature can also embrace terms like isolate-site hydrates (water molecules are isolated from direct contact), channel hydrates (containing chains of water molecules) and ion-associated hydrates (where metal ions are coordinated with water) [31].

This study reports the first hemihydrate form of racemic PZQ (PZQ-HH). Alternative ways for obtaining the new crystal form are explored; namely, a two-step mechanochemical treatment when the commercial PZQ (Form A) is used, or one-step in the case of PZQ Form B. Additionally, PZQ-HH can be also obtained in a one-step mechanochemical process starting from commercial PZQ Form A when seeds of preformed PZQ-HH (5–10% by weight) are introduced in the reaction powder. Finally, by classical slurry method, PZQ-HH can be obtained in three days if starting from Form B. A full characterization of the new solid form (i.e., polarimetry, HPLC, PXRD, SEM, HSM, DSC, TGA, SS-NMR, FT-IR, IDR, saturation solubility, in vitro activity against *S. mansoni* adults, and physical stability in different conditions) is performed, providing important findings for understanding the crystal features and to ascertain the relationship with other PZQ crystal forms.

## 2. Materials and Methods

### 2.1. Materials

Commercial racemic PZQ (polymorphic Form A) of Ph. Eur. grade was a gift of Fatro S.p.A. (Bologna, Italy). Methanol and Ethanol of HPLC grade were purchased from VWR International (Milan, Italy). All the chemicals were used as received, without any further purification.

### 2.2. Methods

#### 2.2.1. Praziquantel Hemihydrate Preparation

The mechanochemical preparation of PZQ-HH and PZQ Form B was performed in an MM400 (Retsch, Germany) vibrational mill equipped by two 35 mL zirconium oxide jars, each one containing two zirconia balls (diameter of 10 mm).

After a set of preliminary trials conducted using different amounts of water and adding water at various stages of the mechanochemical treatment, a two-step method was used for the preparation of PZQ-HH starting from the commercial form of pure PZQ. Specifically, 436 mg of PZQ Form A was initially milled under neat conditions for 30 min at 25 Hz and the intermediate product was assessed by PXRD (results are reported in Figure S1). Subsequently, the product obtained by neat grinding was processed for 1h at 25 Hz in the presence of an equimolar amount of water (corresponding to 22 µL).

The same PZQ-HH could be obtained through a one-step mechanochemical process starting from PZQ Form B. Specifically, 436 mg of anhydrous Form B and an equimolar amount of water (corresponding to 22 µL) were ground for 60 min at 25 Hz in a zirconia jar.

Another method of producing PZQ-HH consisted of the use of preformed seeds of PZQ-HH and PZQ Form A. Specifically, 414 mg of anhydrous PZQ Form A and 22 mg of PZQ-HH (corresponding to a 95:5 PZQ Form A-to-PZQ-HH wt ratio) or 392 mg of anhydrous PZQ Form A and 44 mg of PZQ-HH (corresponding to a 90:10 PZQ Form A-to-PZQ-HH wt ratio) were ground in the presence of 22 µL of water in a zirconia jar for 60 min at 25 Hz.

PZQ polymorphic Form B was prepared using a method already reported in the literature [19]. Briefly, 850 mg of PZQ Form A was milled in neat conditions for 4 h at 20 Hz, and the conversion of PZQ Form A into PZQ Form B was eventually assessed by powder X-ray diffraction, differential scanning calorimetry and FT–IR spectroscopy, using the same conditions previously applied [19].

#### 2.2.2. Slurry Experiments

A large excess of either commercial PZQ (Form A) or Form B was added to 1 mL of distilled water and left under stirring at room temperature for several days (1–7 days) and the solid samples were periodically checked. In particular, after filtration, the excess of solid was immediately analyzed by powder X-ray diffraction.

#### 2.2.3. Differential Scanning Calorimetry and Thermogravimetric Analyses (DSC-TGA)

For the DSC analysis, 2 mg of solid sample was introduced into a 40 μL aluminum perforated crucible and analyzed using a Mettler Toledo DSC822e (Greifensee, Switzerland) from 30–200 °C (10 °C/min) under a nitrogen atmosphere (a flow rate of 50 mL/min). For the TGA analysis about 10–15 mg of the sample was accurately weighted in a 40 μL aluminum crucible. The analyses were conducted with a Mettler Toledo TGA/SDTA851e (Greifensee, Switzerland) using the same conditions as the above DSC procedure.

#### 2.2.4. FT-Infrared Spectroscopy (FT–IR)

The samples were analyzed with a Perkin-Elmer System 2000 FT–IR instrument (Norwalk, CT, U.S.) in a range of 400–4000 cm^−1^ after being gently ground in an agate mortar with anhydrous KBr (in a 1:15 ratio by wt) and pressed into a disc with a hydraulic press. The resolution used was 4 cm^−1^ with a step of 1 cm^−1^ and a scan number of three.

#### 2.2.5. Polarimetry and Drug Recovery

The optical rotations of the samples dissolved in ethanol (1 g/100 mL) were recorded at 25 °C, λ = 589 nm using a Jasco P2000 Polarimeter (Lecco, Italy), according to a method already reported [9,19,32]. The use of ethanol instead of CHCl_3_ permitted an easier preparation of the samples without air bubbles in the cell.

For the determination of drug content, the HPLC apparatus was an Agilent HPLC-UV 1260 Infinity II (Santa Clara, CA, US) with a column EC-C18 Poroshell 120 Å of 4 µm and the dimensions of 4.6 × 10 mm. The analyses were performed at a controlled temperature of 25 °C, with a mobile phase composed of methanol:water (65:35 v/v) and with a flux of 0.750 mL/min. A fixed wavelength of 220 nm was used for detection. The peaks were integrated using the external standardization method for quantification. The total run time was 10 min and PZQ retention time was about 7.7 min. Prior to sample analyses, a calibration curve of PZQ in the range of 0.5–10 mg/L was established (r^2^ of 0.9988). Each time before the analysis a standard solution was prepared by dissolving 10 mg of PZQ in 20 mL of methanol and analyzed in the HPLC system after dilution with the mobile phase (1:200). The standard solution had a concentration of 2.5 mg/L.

#### 2.2.6. Hot Stage Microscopy (HSM)

A hot stage microscopy apparatus (Mettler-Toledo Ltd., (Greifensee, Switzerland) was used for the observation of the samples by taking movies upon heating from 25–150 °C using a dedicated ocular and software. The images were collected through a micro ocular MD-300 and Webcam Companion software.

#### 2.2.7. Powder X-ray Diffraction

Powder X-ray diffraction routine analyses were performed using a Panalytical X’Pert Pro Diffractometer (Panalytical, Almelo, Netherlands) composed of a RTMS X’celerator detector and with Cu Kα radiation without monochromator (1.5418 Å). The analyses were conducted in a 2θ range of 3°–40° with a step size of 0.0334° and a scan speed of 0.142°/s. Each sample was prepared by gently pressing a sufficient amount of powder with a glass slide into the cavity of a sample holder to give a flat surface.

Data collection for the purpose of crystal structure determination of PZQ-HH was performed on a capillary powder diffractometer. The powder sample was mounted in a 0.3 mm borosilicate glass capillary and rotated in the beam during collection. A STOE STADIP transmission diffractometer, and a Mythen1k detector with an 18° 2θ angular range was used. The pattern was collected at RT temperature using a monochromatic Cu Kα1 radiation (1.54056 Å) at 40 kV, 30 mA, from a focusing Ge(111) primary beam monochromator, from 2°–70° 2θ, stepping at 0.1° and 15 s per step.

#### 2.2.8. Scanning Electron Microscopy

Form A, Form B and the hemihydrate were observed using a JEOL JSM-5510LV scanning electron microscope (Welwyn, UK) after being metalized with gold with a sputter coater. Selected samples were observed by environmental scanning electron microscopy using a Quanta 200 FEI-XRF (Felmi-ZFE, Graz, Austria) embedded instrument.

#### 2.2.9. Solid-State NMR

Solid-state NMR measurements were acquired on a Jeol ECZR 600 instrument (Akishima City, Tokyo, JP), operating at 600.17 and 150.91 MHz for the ^1^H and ^13^C nuclei, respectively. Powder samples were packed in 3.2 mm diameter cylindrical zirconia rotors with the volume of 60 µL. A certain amount of sample was taken and used without further preparation from each batch to fill the rotor. ^13^C CPMAS spectra were acquired at a spinning speed of 20 kHz with a RAMP-CP pulse sequence (90° ^1^H pulse of 2.2 µs; contact time of 3.5 ms), a recycle time of 38.1 s and 280 scans. The two-pulse phase modulation (TPPM) decoupling scheme with a 119.0 kHz radiofrequency field was used during the acquisition period. ^13^C chemical shifts were referenced to α-glycine (^13^C methylene signal at 43.5 ppm).

#### 2.2.10. Crystal Structure Determination from Powder X-ray Diffraction Data

The PXRD pattern of PZQ hemihydrate material was indexed using the N-TREOR algorithm [33] via an interface of EXPO2014 [34]. The indexing procedure revealed a triclinic unit cell with the volume of 860.2 Å^3^, which corresponds to two PZQ molecules per unit cell. Since the hemihydrate material was prepared from a racemic anhydrous PZQ, the structure solution was attempted in a centrosymmetric P-1 space group. The structure was solved using the simulated annealing procedure implemented in EXPO2014 [34]. The asymmetric unit contained one PZQ molecule and one oxygen atom corresponding to one water molecule. Structure solution processing involved a large number of randomized steps where translational (for both PZQ and water oxygen) and rotation (PZQ only) degrees of freedom were varied. In addition, intramolecular rotation around the flexible bonds was allowed. The PZQ fragment was given full occupancy, while the water oxygen occupancy was fixed at 0.5, reflecting the experimentally determined stoichiometry of the material. Rietveld refinement [35] of the structure was performed in TOPAS Academic 4.1 [36]. In addition to the translational, rotational and intramolecular degrees of freedom, zero-point shift, peak shape function and background polynomial were refined. The diffraction peak shape was described by a pseudo-Voigt function, while the background was fitted with a 12-term Chebyshev polynomial function. The occupancy parameter of the water oxygen atom was allowed to refine, and the occupancy remained close to the expected value of 0.5. In the final refinement, oxygen occupancy was once again constrained to 0.5, and two hydrogen atoms were inserted in the most probable positions, corresponding to the hydrogen bond directions towards the carbonyl oxygen of PZQ. The resulting structural model reveals that the water molecule is disordered over an inversion center with a 50:50 chance. Crystallographic parameters of the hemihydrate structure are summarized in Table S1.

#### 2.2.11. Periodic DFT Calculations

Periodic DFT calculations were performed using the plane-wave DFT code CASTEP 16.1 [37]. Crystal structures of polymorphs of anhydrous PZQ (A and B), as well as the racemic hemihydrate structures, were geometry optimized with the aim of calculating the relative stability of these crystal forms. The experimental crystal structures were converted in CASTEP format with the help of cif2cell [38] utility. The DFT calculations were performed using semi-local PBE [39] functional combined with a Grimme D2 [40] semiempirical dispersion correction. The plane wave basis set was truncated at a 700 eV cutoff, and the norm-conserving pseudopotentials were used to modify the Coulomb potential in the core regions of electron density. The electronic Brillouin zone was sampled with a 0.03 Å-1 Monkhorst-Pack k-point grid [41]. Geometry optimization involved variation of atom coordinates and unit cell parameters subject to the symmetry constraints of the corresponding space groups. The following convergence criteria were used: maximum energy change 10^−5^ eV per atom, maximum force on atom 0.01 eV Å^−1^, maximum atom displacement 0.001 Å and residual stress 0.05 GPa.

The DFT-optimized crystal structure was used to assess the accuracy of experimental crystal structure determination from PXRD data. The overlay between the optimized and experimental structures revealed a root mean square Cartesian displacement (RMSCD) value of 0.136 Å, which is well within the limits of what is considered a correct structure determination [42].

#### 2.2.12. Modeling of Solid-State NMR Spectra

The optimized crystal structures were used for modeling solid-state NMR spectra. NMR parameters were calculated using the CASTEP implementation of GIPAW method [43]. The calculations used PBE semi-local functional, with a plane-wave basis set truncated at a 1000 eV cutoff. Core regions of electron density were described using on-the-fly generated ultrasoft pseudopotentials [44]. The calculated chemical shieldings were converted into chemical shifts using a reference shielding of 170 ppm. The spectral lines were drawn with Lorentzian curves with 1 ppm HWHM.

#### 2.2.13. Saturation Solubility and Intrinsic Dissolution Rate (IDR)

The solubility of the samples in water was analyzed by preparing 10 mL of saturated solutions of each sample in deionized water that were kept in the dark under agitation for 48 h until equilibrium. Then, the solutions were filtered using a membrane (pore size = 0.45 μm) and diluted 1:200 with the mobile phase prior to injection into the HPLC system. The HPLC methodology used for the quantification was reported in Section 2.2.5. For the intrinsic dissolution rate determination, about 150 mg of the samples were inserted in the sample holder and pressed using a hydraulic press (PerkinElmer, Norwalk, U.S.) for 1 min at 1 ton. No solid-state transitions have occurred due to compaction under these conditions, as verified by PXRD analyses (Figure S2). The sample surface area obtained was of 0.785 cm^2^ and the entire sample holder with the compressed powder was immersed in a vessel containing 1 L of distilled water kept at 37 °C. The system used was a Hanson Research SR8 Plus (Chatsworth, CA, U.S.) dissolution test station and the paddles were positioned 3.5 cm from the tablet surface, with a rotation speed of 100 rpm. About 2 mL of the dissolution medium were withdrawn every ten minutes until 60 min and immediately replaced with an equal amount of thermo-stated distilled water. The aliquots were then diluted 1:20 with the mobile phase and analyzed using the same above-mentioned method. The analyses were performed in triplicate and for each point and the mean with SD (%) and RSD (%) were calculated. The amount of dissolved drug per unit area over time was indicated by the slope of the curves, obtained through a linear regression method (r^2^ > 0.99 in all cases). For the comparison of the different curve the one-way ANOVA test was used, and the data were considered statistically different where the *p* value < 0.05.

#### 2.2.14. In Vitro Activity

The in vitro activity of PZQ-HH was tested using adult *S. mansoni*. The study was approved by the local veterinary agency, based on Swiss cantonal and national regulations (permission no. 2070). At least three adult worms (both sexes) obtained from dissecting *S. mansoni* infected mice were incubated with RPMI 1640 (Gibco, USA) supplemented with 5% (v/v) fetal calf serum (FCS) and 1% (v/v) pen–strep at 37 °C, 5% CO_2_ for 72 h for each concentration tested ranging from 0.021–0.33 μg/L. By using an inverse microscope (Carl Zeiss, Germany, magnification 80×) and viability based on all the alterations of morphology, motility and viability were recorded and the IC_50_ value was calculated with CompuSyn software (ComboSyn Inc., Paramus, NJ., U.S.).

#### 2.2.15. Physical Stability under Several Conditions

Several batches of PZQ-HH were kept at room temperature in closed containers, protected from light over a period of several months. Its physical state was periodically analyzed using PXRD.

Further, PZQ-HH physical stability upon thermal and mechanical treatment was tested. In particular, PZQ-HH was heated at a constant temperature of 50 °C under vacuum over-night and the obtained product was assessed by means of PXRD. PZQ-HH was subjected to 30–80 °C dynamical heating and analyzed by in situ PXRD. Finally, PZQ-HH was ground for 60 min at 25 Hz and again assayed by PXRD.

## 3. Results

### 3.1. Mechanochemical Preparation of PZQ-HH Using PZQ Form A as Starting Material

Initially, PZQ Form A was processed through liquid assisted grinding (LAG) in the presence of water. The solid products recovered from these experiments did not present structural changes and pure anhydrous PZQ Form A was the only solid form observed. Further trials conducted by varying milling frequency, time or water amount also resulted in pure anhydrous PZQ Form A. Slurry experiments in water also produced anhydrous PZQ Form A, i.e., the same polymorph as the starting PZQ. Representative PXRD patterns of some products are reported in Figures S3 and S4. The nature of the polymorphs in the solid product at the end of each process is summarized in Table 1.

Conversely, a new solid form was obtained when operating in a two-step mechanochemical method. In particular, Form A was initially milled in neat conditions for 30 min at 25 Hz. As assessed by PXRD (Figure S1) the product obtained at this point is a low-crystallinity material, with a remarkable amorphous halo in the background, with some residual peaks of Form A and some signals attributed to Form B. Subsequently the grinding process was stopped to introduce water and restarted for a further 60 min at 25 Hz. It is worthy of notice that also, in this case, every change in the quantity of water inserted led to the same solid form: after 60 min of grinding in the presence of water, the complete transformation to the new solid phase was achieved. The material obtained from this grinding condition was a racemic hemihydrate, that will be later described and fully characterized.

**Figure 2:**
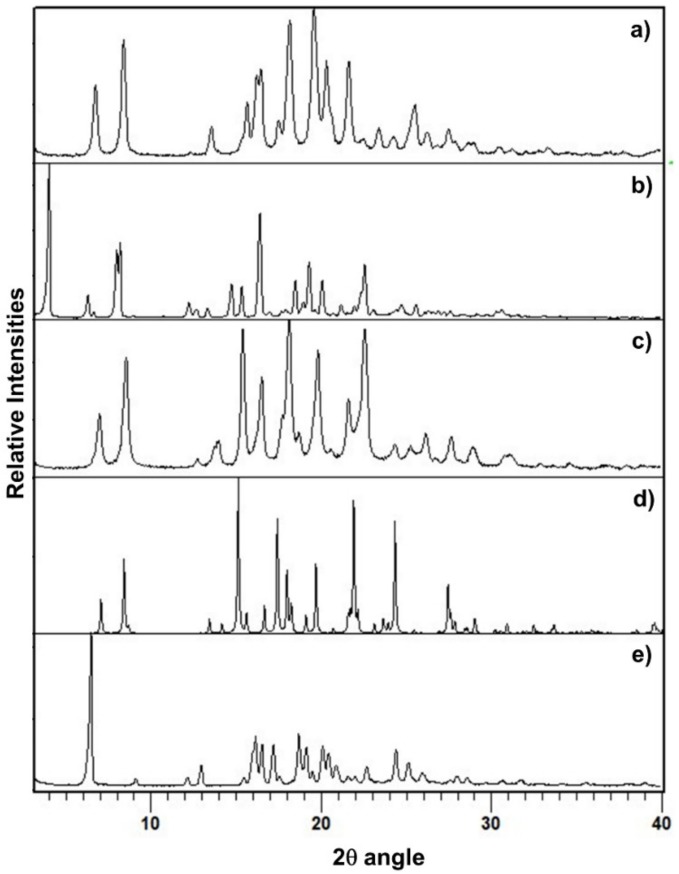
PXRD pattern of (**a**) Form B, (**b**) Form A, (**c**) Form C, (**d**) the calculated pattern from CSD of the enantiomeric hemihydrate SIGBUG and (**e**) the new racemic hemihydrate.

The PXRD pattern of PZQ-HH was completely different from the already known PZQ structures, as shown in Figure 2, in which the reader can also find the calculated pattern of the enantiomeric hemihydrate (indexed as SIGBUG [15] in the CSD), Form B, Form C and Form A; the characteristic reflections of PZQ-HH were found at 6.28°, 16.14°, 16.50°, 17.18°, 18.67°, 19.12°, 20.05°, 20.41° and 24.37° in 2θ.

### 3.2. Mechanochemical Preparation of PZQ-HH Using PZQ Form B as Starting Material

When PZQ Form B was used as a starting material, PZQ-HH formed in a one-step process and the preliminary neat grinding stage was not necessary. Additional LAG experiments using different amounts of water led to the same solid form, suggesting that, in the present case the amount of water does not drive the formation of a hydrate with higher water stoichiometry. Since it has been reported that, in a given solvate-forming system, it is possible to access different stoichiometric forms by simply changing the amount of liquid during the mechanochemical formation of solvates [45], it can be speculated that in the case of PZQ and water the only (energetically) accessible stoichiometry is the 1:0.5 molar ratio (hemihydrate). Slurry experiments of Form B in water also gave PZQ-HH (Table 1, Figure S4).

### 3.3. Mechanochemical Preparation of PZQ-HH Starting with PZQ Form A and Seeds of Preformed PZQ-HH

**Figure 3:**
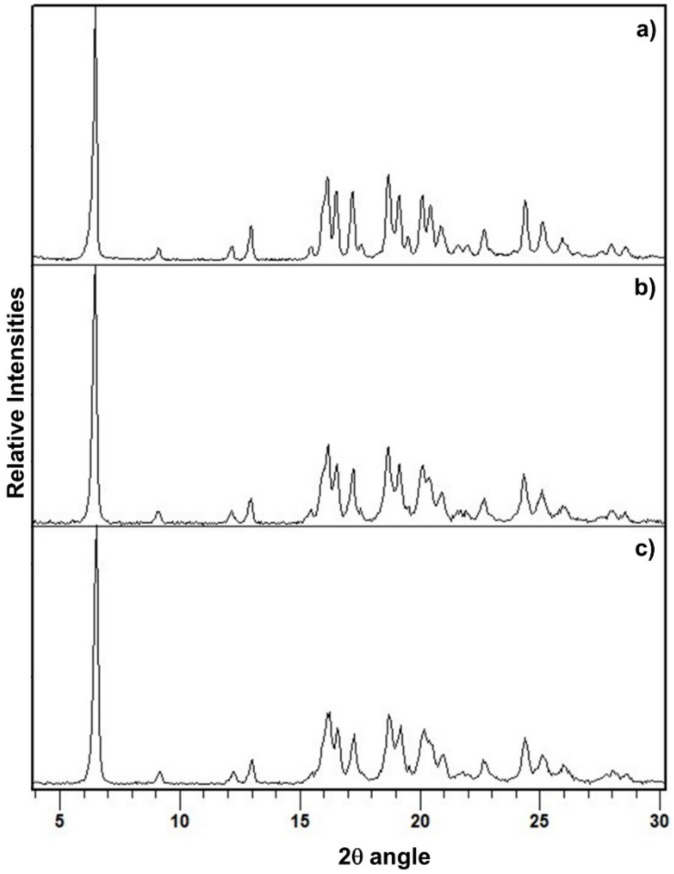
PXRD patterns of (**a**) PZQ-HH obtained in a one-step process starting from Form B, (**b**) PZQ-HH obtained in a one-step process by seeding procedure (with 10% wt preformed PZQ-HH) starting from Form A and (**c**) PZQ-HH obtained in a one-step process by seeding procedure (with 5% wt preformed PZQ-HH) starting from Form A.

When PZQ Form A/PZQ-HH (95:5 or 90:10 by wt) mixtures were used as a starting powder, after 60 min of grinding at 25 Hz in the presence of water, complete transformation of the solid in PZQ-HH was achieved (Table 1, Figure 3). A one-step process in the presence of seeds of preformed PZQ-HH was hence enough to promote the formation of PZQ-HH and the preliminary neat grinding stage was not necessary.

### 3.4. Characterizations of PZQ-HH and Crystal Structure Solution

To fully comprehend the often complex behavior of a mechanochemical-prepared hydrate a multidisciplinary investigation is paramount. This would include chemical, thermal, structural, spectroscopic, morphological, biopharmaceutical and stability evaluations. Since this is a new solid hydrated form, a thorough characterization of PZQ-HH was carried out and phase interrelations to the known PZQ polymorphs were studied.

#### 3.4.1. Chemical Analyses

To ensure the racemic nature of PZQ in the hemihydrate, polarimetric analyses were performed on different samples and compared to Form A: the mean [α]_λ_ value registered for PZQ-HH was 0.02 ± 0.06, perfectly corresponding to a racemic compound, as in the case of the commercially available Form A ([α] of 0.5 ± 2.9). HPLC results showed a complete drug recovery after mechanical treatment, attesting the lack of chemical changes in PZQ. This means that when PZQ is ground in the presence of little amounts of water (equimolar) does not degrade, similarly to what was noted when grinding PZQ alone [19,20], but unlike what was seen in the presence of polymeric excipients [22,23,24]. This also highlights that the presence of water, at least in little amounts during milling does not favor chemical degradation, even though water is known to be a factor affecting PZQ chemical stability [22]. It is worth noting that no mechanochromism phenomenon was noticed at the end of the process and the final product was a white powder.

#### 3.4.2. Crystal Structure Solution

**Figure 4:**
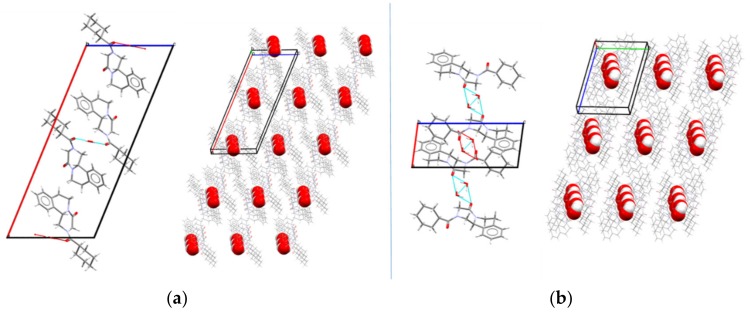
Packing and hydrogen bonds motifs between PZQ and water molecules in (**a**) the enantiomeric hemihydrate SIGBUG [15] and (**b**) in the new racemic hemihydrated form.

The crystal structure of PZQ-HH was solved from the capillary powder X-ray diffraction pattern following the method previously described (paragraph 2.2.10): the space group P-1 of PZQ-HH was the same as Form A; however, 1 molecule is present in the asymmetric unit of PZQ-HH, rather than 4, as in TELCEU. Compared to the enantiomeric hemihydrate (SIGBUG), in this case, every water molecule in PZQ-HH is connected with two PZQ entities, one R- and one S-, through hydrogen bonds, as reported in Figure 4.

**Figure 5:**
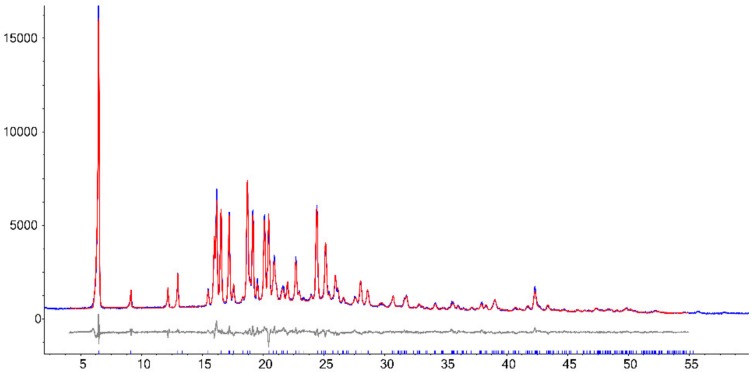
Rietveld refinement fit of the experimental pattern (red) and the calculated one (blue). In grey are the corresponding residuals.

The Rietveld refinement fit of the experimental pattern with the calculated one is reported in Figure 5. The refined unit cell had the following parameters: space group P-1, cell lengths (Å) of a = 5.857, b = 10.921 and c = 14.299; cell angles of α = 105.755°, β = 94.622°, γ = 99.564°; volume (Å^3^) of 860.342; density of 1.24 g cm^–3^.

PZQ does not have any H-bond donor group, thus these interactions are possible thanks to the presence of the oxygen atoms acting as acceptors since the nitrogen electron pair is engaged in the electron delocalization, as it was also reported in the literature [17]. Observing the packing of the unit cell of the new racemic hemihydrate and the enantiomeric one (Figure 4), in both cases the PZQ molecules are inversely positioned and linked to the water molecule between them. The difference is in the oxygen involved in such bonds: in PZQ-HH the molecules are linked via an Ow-H···O=Cet hydrogen bond, while in the enantiomeric hemihydrate the oxygen bonded to the water is the cyclohexylic one (the oxygen atom linked with the cyclohexyl group), creating an Ow-H···O=Ccy hydrogen bond. Concerning conformation, while the starting PZQ has the *syn* conformation, in the PZQ-HH *anti*-conformation was observed. Of note, the same *anti*-conformation was found in both Form B and C [19,20]. Moreover, the water molecules are not linked to each other but are instead connected with the drug molecule, meaning that the new PZQ-HH belongs to the isolated-site hydrates [31,46].

#### 3.4.3. Thermal Analyses

**Figure 6:**
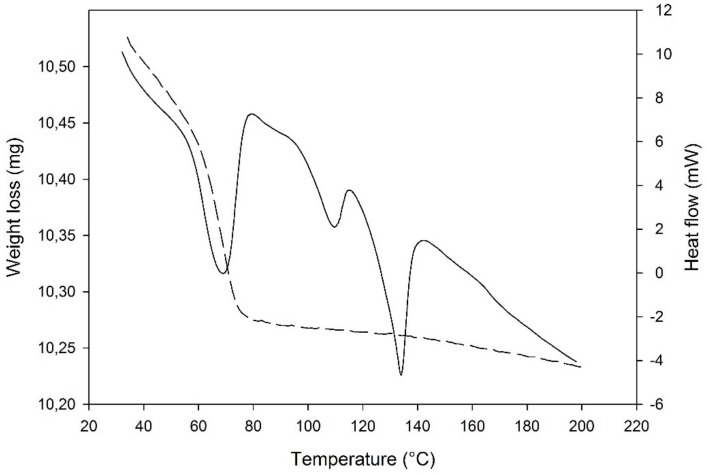
DSC-TGA traces of PZQ-HH (solid lines refer to DSC curve).

The DSC curve (Figure 6) presented a sharp dehydration endotherm in a narrow weight loss range, in comparison to other solid systems containing water: this was expected for isolated site hydrates as the new PZQ-HH, according also to the literature [46]. This sharp endothermic dehydration at about 68 °C (61.38 J/g) was confirmed by weight loss in the TGA, which was 2.19% and in good agreement with the theoretical value of 2.73% for a hemihydrate. Notably, at the TGA the water loss started from the beginning of the analysis at room temperature. Other endothermic events were recorded at the DSC at 109.05 °C and 133.95 °C with an enthalpy of fusion of 10.25 J/g and 32.49 J/g that were identified as melting events of Form B [19] and Form A [22], respectively.

Hot stage microscopy analysis shows that PZQ-HH collapses at around 68–75 °C, forming a liquid phase. This event is followed by a partial recrystallization of Form B and its melting at around 110 °C [19], after which a partial recrystallization of the native Form A can be seen ending in the final melting around 135–138 °C, as reported in Figure S5.

#### 3.4.4. Experimental and GIPAW-DFT Calculated ^13^C CPMAS SS-NMR Spectra

**Figure 7:**
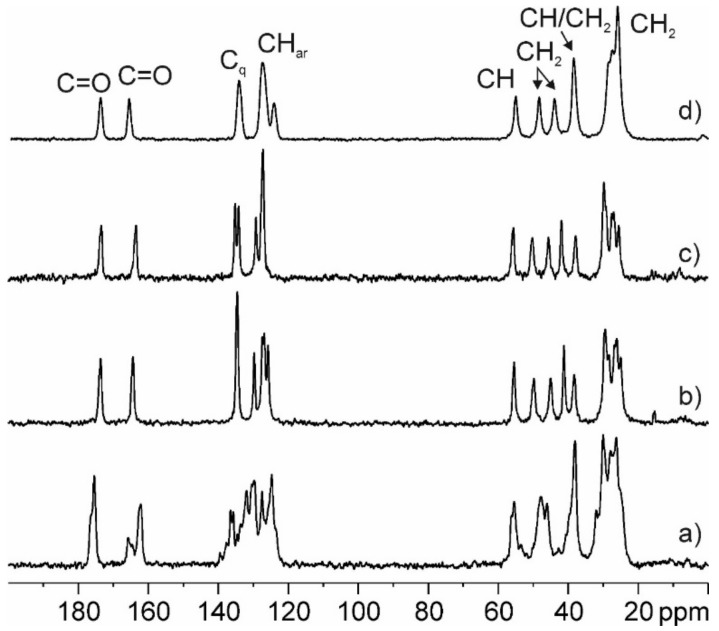
^13^C (150.9 MHz) CPMAS spectrum of PZQ-HH (**d**) recorded at 20 kHz with principal group assignments in comparison with previously reported spectra of commercial PZQ (**a**) Form B (**b**), and Form C (**c**).

The ^13^C CPMAS SS-NMR spectrum of PZQ-HH was compared to the anhydrous polymorphs Form A, B, and C reported in Figure 7. The analysis confirmed the formation of a new pure phase different from the previous, the presence of only one molecule in the asymmetric unit (due to the single set of signals compared to the multiple ones of Form A) and a lower crystallinity of the sample (FWHM ~150 Hz) with respect to the other forms (FWHM ~90–100 Hz). Also, the experimental data match the GIPAW-DFT calculated one, as reported in Figure S6 and in Table 2 compared to that of Form A, B, and C, with a root mean square error of 1.8 ppm. This overall value represents the agreement between the computed ^13^C chemical shifts of the powder X-ray structure and those obtained experimentally, and it is much better than those reported in the literature for correct structures (around 2.2 ppm) [47]. This confirms the reliability of the structure solved from PXRD data.

#### 3.4.5. FT–IR Spectroscopy

**Figure 8:**
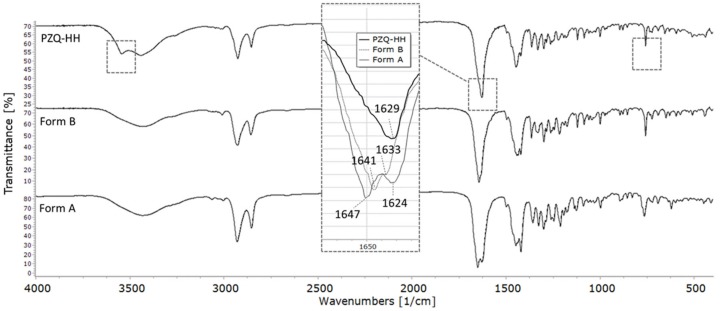
FT–IR spectra of (**top**) PZQ-HH, (**middle**) Form B and (**bottom**) Form A. Main differences in comparison to the anhydrous forms are indicated in the frames.

The FT–IR spectrum of PZQ-HH, compared to pure PZQ Form A and Form B, is shown in Figure 8. Differently from the anhydrous forms, PZQ-HH presents a sharper OH band at 3543 cm^−1^ (indicated by the frame). This sharp OH stretching band is also in accordance with the previously mentioned isolate class of hydrates [46]. In the typical range of C=O stretching (see the frame in Figure 8), a single broad band is observed at 1629 cm^−1^ of PZQ-HH, confirming the intermolecular interaction between the drug and water via PZQ carbonyl groups. It also shows the C=Oet is shifted by about 22 cm^−1^ in comparison to Form A, while the C=Ocy, involved in the hydrogen bond, is not visible. The signal at 758 cm^−1^ (highlighted by the frame), corresponding to the bending of the aromatic –CH, is superimposable to that of Form B, whilst it was at 765 cm^−1^ for Form A. This is in agreement with previously reported SS-NMR assignments for Cq attesting high similarities between Form B and PZQ-HH, while having significant differences from Form A.

#### 3.4.6. Morphological Analyses

**Figure 9:**
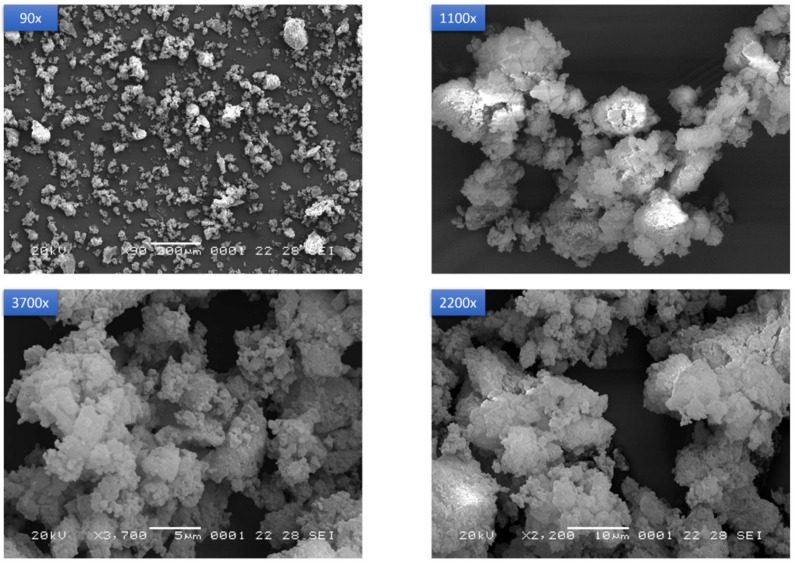
SEM images of PZQ-HH powder at different magnifications (90×, 1100×, 2200× and 3700×).

From electron microscopy analyses, the new solid form consisted of agglomerates of large plates, assuming a porous aspect with a broad specific surface area (Figure 9). The particle size of the powder varies in the range 40–100 µm. This morphology is clearly distinguishable from the reported needle-shaped particles of starting Form A [22], from the whiskers in Form B and Form C clusters of particles, even though these latter were also obtained by mechanochemistry [19,20].

#### 3.4.7. Saturation Solubility, IDR and Antischistosomal Activity

**Figure 10:**
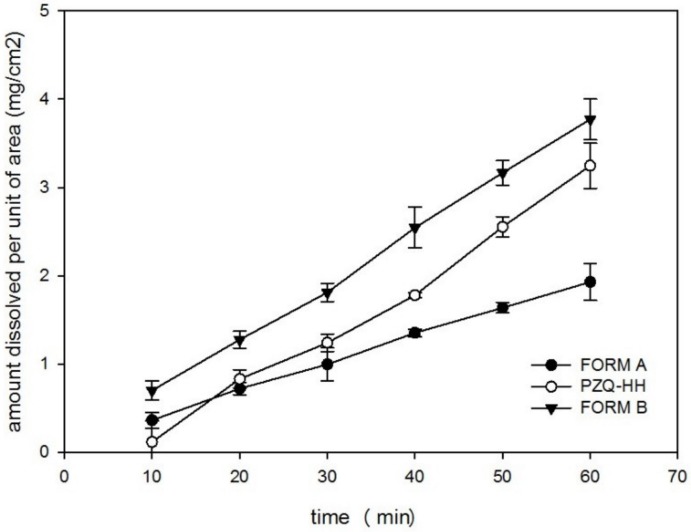
Intrinsic dissolution rate profiles in water at 37 °C for PZQ-HH, Form B and Form A.

PZQ-HH demonstrated excellent biopharmaceutical properties, since the water solubility (after 48 h at 25 °C) was of 310.89 ± 3.07 mg/L, while for Form A was 217 ± 10.33 mg/L. The intrinsic dissolution rate (IDR), as reported in Figure 10, was twice that of the commercially available Form A. In fact, a value of 0.0618 ± 0.0051 mg/cm^2^/min was found for PZQ-HH, while the raw drug showed an intrinsic dissolution rate of 0.0299 ± 0.031 mg/cm^2^/min. A similar feature was attested for Form B, as previously reported [19], and the statistical comparison between the Form B value and that of the new hemihydrate did not reveal any significant difference, which was conversely detected when comparing the IDR of PZQ-HH with the one of Form A.

The hemihydrate form was assayed for its activity in vitro against *S. mansoni* adults: it exhibited an IC_50_ of 0.15 μM, identical to commercially available Form A (IC_50_ of 0.1. μM) [48].

### 3.5. Physical Stability under Various Conditions

**Figure 11:**
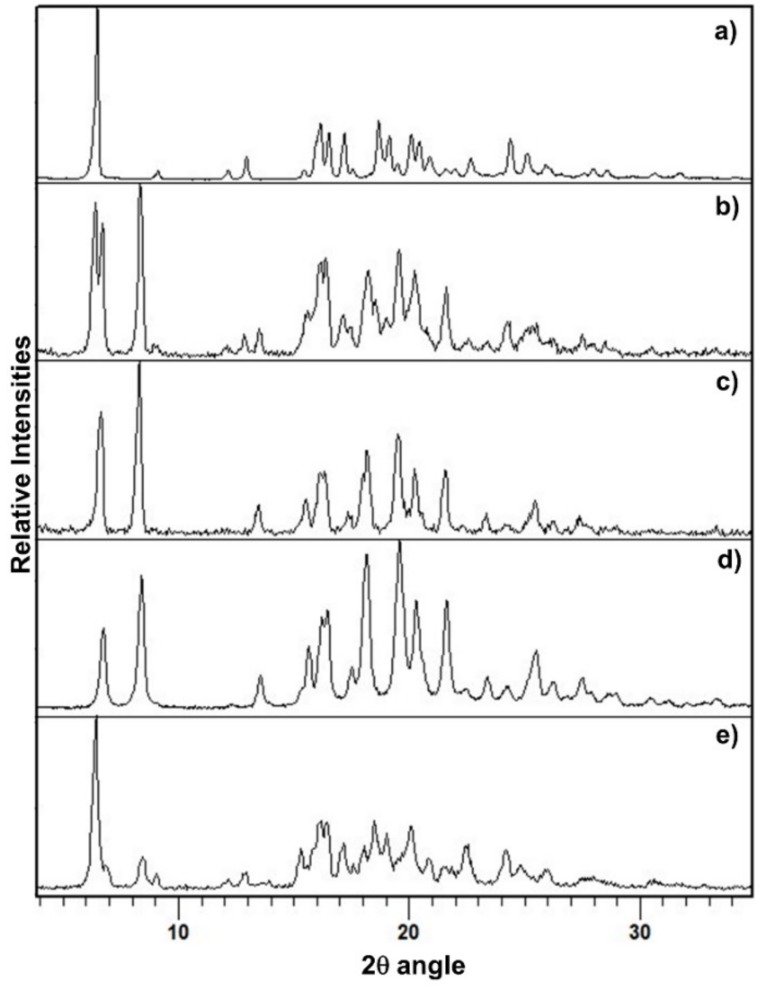
PZQ-HH progressive transformation to Form B at room temperature (from top to bottom): (**a**) fresh sample, (**b**) after 4 months, (**c**) after 12 months, (**d**) Form B, and (**e**) PZQ-HH ground for 30 min at 25 Hz.

PZQ-HH, kept at ambient temperature in closed vials, was physically stable for three months. After this period, PXRD revealed that Form B started appearing, while there were no signals referable to Form A (as visible in Figure 11).

Milling of PZQ-HH slightly decreased peak intensities and after 60 min at 25 Hz the PXRD pattern showed a complete transition from PZQ-HH to Form B (Figure 11 reports the intermediate product at 30 min).

**Figure 12:**
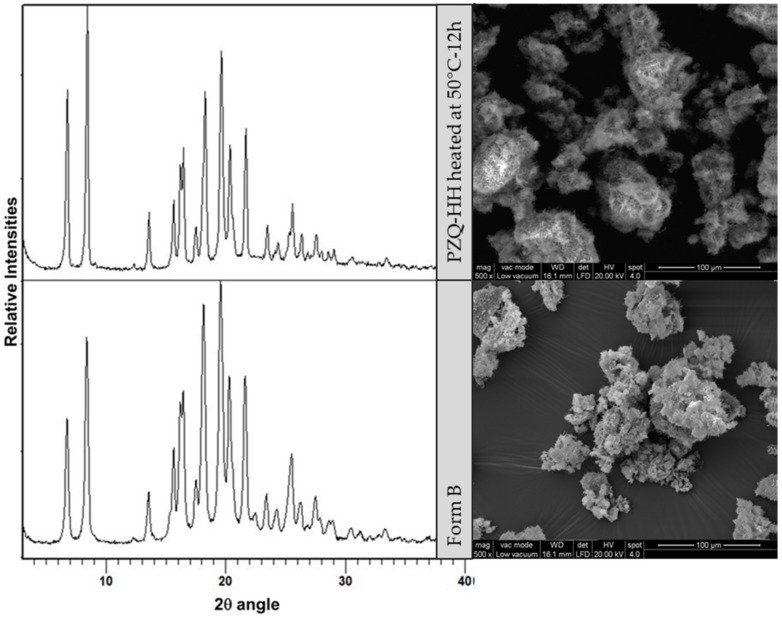
PXRD patterns (**left**) and magnified microscopy images (**right**) of PZQ-HH heated overnight at 50 °C/35 mmHg (**top**), and Form B (**bottom**).

To check stress-induced transformations, PZQ-HH was left at 50 °C, under vacuum (35 mmHg) overnight. Again, Form B was obtained as demonstrated by PXRD and ESEM analyses (both reported in Figure 12). In particular, the habitus of the dehydrated product sample was very similar to that of Form B, with typical agglomerates of whiskers.

Finally, variable-temperature in situ PXRD was carried out for PZQ-HH, to online monitor changes of the solid product upon heating. The results, reported in Figure S7, confirm a gradual dehydration and a complete transformation to Form B at 60 °C. Whereupon anhydrous Form B remained stable until the end of the analysis (80 °C).

Once again we address the reader to Table 1 for the nature of the polymorphs detected in the solid product at the end of each process.

To assess the thermodynamic aspects of transformations of crystal forms of PZQ we have compared the energies of the structures obtained in periodic DFT calculations. The racemic PZQ-HH phase has been found as 1.8 kJ mol^−1^ lower in energy than the known enantiomeric hemihydrate phase (CSD SIGBUG, SIGBUG01). The lower energy of the racemic phase explains its preferential formation from the racemic PZQ, as opposed to a 50:50 conglomerate of R and S crystals of the enantiomeric form.

In addition to comparing the lattice stabilities of the two hemihydrate phases, we have calculated the dehydration energy of PZQ-HH towards the anhydrous polymorphs (A and B) of PZQ. In the case of anhydrous PZQ Form B the process was found to be endothermic with an energy of +39.4 kJ mol^–1^. For comparison, the dehydration energy for the formation of anhydrous Form A was found to be less endothermic at +31 kJ mol^–1^. This confirms that Form B is a metastable polymorph, the formation of which under experimental conditions must be explained by kinetic, rather than thermodynamic factors.

## 4. Discussion

The present study shows that the identity of PZQ crystal form used as the starting material in the mechanochemical formation of PZQ-HH affects the course of the reaction. As mentioned above, PZQ-HH can only be obtained from anhydrous Form A via a two-step grinding mixed process using initially neat and subsequently LAG conditions in water. The first neat step involves the formation of a mainly amorphous intermediate. Amorphous solids are usually very hygroscopic, and the presence of moisture is a key factor for recrystallization. Recrystallization of the amorphous intermediate product as a hemihydrate is therefore expected since the kinetic barrier is significantly reduced. Hence, if water is added to this mixture in a subsequent step of grinding, after the formation of the amorphous intermediate, the reaction evolves to the formation of PZQ-HH. It is noteworthy, and rather counterintuitive, that milling PZQ Form A directly with water results in no hydration. Differently, single-step LAG of PZQ in water results in the hemihydrate when the reactant is PZQ Form B, possibly related to the structural similarity between the two forms, as better addressed below. The present findings add to the recent studies involving real-time in situ grinding experiments, which show that mechanochemical reactions may present different mechanistic steps and intermediates [49,50,51].

**Figure 13:**
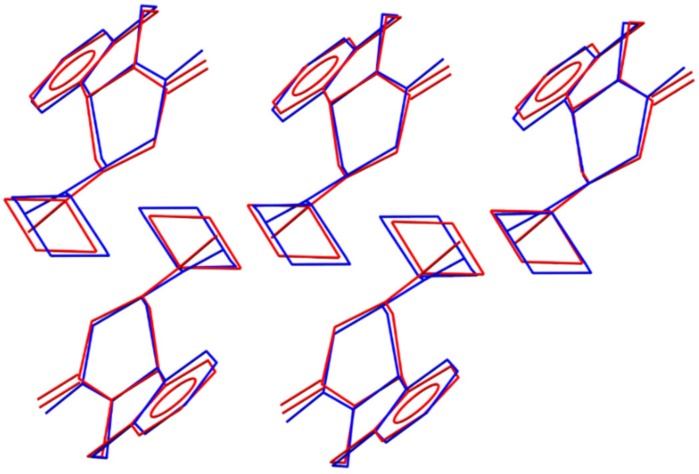
Overlay of the PZQ-HH structure (**blue**) and PZQ Form B (**red**) showing a cluster of five matching PZQ molecules. Hydrogen atoms and positions of water molecules in the PZQ-HH structure were omitted for clarity.

A classic example of how the nature of the starting materials plays a role in the mechanochemical reaction outcome is the case of the cocrystallization of caffeine and citric acid [52]. The case of PZQ-HH, on the other hand, is special not because of the presence of water in the reaction powder per se but because of the structural differences between the conformation of PZQ molecules in the lattice of Form A (*syn*) compared to the conformation in Form B and the hemihydrate (*anti*). As presented in Figure 13, PZQ-HH and anhydrous PZQ Form B present a high similarity in their crystal structures, which goes beyond the molecular conformation. We speculate that the activation energy required to rotate the molecules into different conformers is a high kinetic barrier for this mechanochemical reaction. This hypothesis also explains why the preparation of PZQ-HH via LAG with water is a direct process either when PZQ Form B is in the reaction vessel or when Form A is seeded with preformed PZQ-HH prior to grinding. The presence of seeds offers an energetically accessible template on which to grow the new solid phase [53], driving the product towards the seeded hydrated form. Similarly, it is likely that the surfaces of Form B particles provide this energetically accessible support, which overcomes the kinetic barrier related to conformer switching [19].

The slurry experiments also confirm this previous hypothesis, provided that slurrying Form A in water results in unchanged material throughout the experiment. It shows that the formation of PZQ-HH is kinetically hindered when starting from Form A regardless of the maximum water activity in solution. Differently, when PZQ Form B is subjected to slurry experiments, the *anti*-conformer may facilitate the interaction and the transformation into the new hemihydrate phase.

The relationships between PZQ anhydrous polymorphs and the hemihydrate were also assessed from the dehydration of PZQ-HH under different environmental conditions. The reaction follows the Ostwald rule of stages as PZQ-HH dehydrates into the metastable Form B, rather than the most stable Form A [54]. In this case, neither during storage under room conditions, nor during mechanical or thermal treatments was there evidence of a direct transition to the stable Form A. As for the relationship among PZQ-HH and Form C, in no case was the formation of the polymorph C observed from the hemihydrate. Once again, the similar packing arrangement of PZQ molecules in Form B explains the formation of this polymorph as a kinetically-preferred dehydration product, even though Form A would be the thermodynamically most favorable product. The *syn-anti*-conformational modification might be the source of the energy barrier that hinders the formation of Form A from dehydration. A possible polymorph interconversion, previously noticed with other single and multicomponent polymorphic systems either through neat [55] or LAG [56] can be hypothesized also in the PZQ-HH/Form B pair. In terms of solubility and IDR, PZQ-HH showed an unusual and favorable biopharmaceutical performance, which is superior to that of anhydrous Form A. PZQ-HH can hence be part of the list of peculiar hydrates, together with the well-known erythromycin dihydrate [57] and tranilast [58]. Those crystal forms counteract the general rule that an anhydrous form is usually more soluble in water than the hydrated form. The enhancement in drug solubility and intrinsic dissolution rate of PZQ-HH can be explained because the water molecules may act as a wedge pushing the PZQ molecules apart, challenging their interaction in the crystal and weakening the structure. Additional reasons for the solubility behavior of PZQ-HH can be hypothesized. From one side, the *anti*-conformation creates wider voids between the molecules which have proven to favor the solubility enhancement of Form B [19] and Form C [20]. Secondly, PZQ has a higher propensity to interact via hydrogen bonds when in an *anti*-conformation. Last but not least, one can expect a crystal form modification when PZQ Form B is in contact with water. In fact, the saturation solubility and dissolution profiles of PZQ-HH and Form B were almost identical between the two forms, suggesting a possible very rapid conversion. These facts were confirmed by the observation of the same conversion of PZQ-HH into the Form B during storage at room temperature/pressure after three months, and after few hours upon heating under vacuum, and finally by the in situ PXRD upon heating. This evidence underlined the close relationship between Form B and PZQ-HH, despite the existence of Form A and C, and the ease of transition from one phase to the other.

## 5. Conclusions

White bulk powder of racemic praziquantel hemihydrate was prepared for the first time, by a mechanochemical method. The hemihydrate structure was solved from PXRD pattern and validated by periodic-DFT calculations: the refined unit cell had the following parameters: space group P-1, cell lengths (Å) of a = 5.857, b = 10.921, c = 14.299; cell angles of α = 105.755°, β = 94.622°, γ = 99.564°; volume (Å^3^) of 860.342; density of 1.24 g·cm^−3^. Due to the novelty of this racemic praziquantel hemihydrate, a complete physico-chemical characterization was performed, by using HPLC, DSC, TGA, HSM, PXRD, SEM, FTIR, polarimetry, solid-state NMR, solubility and intrinsic dissolution rate, and in vitro tests on *S. mansoni* adults. These analyses provided important findings for understanding the crystal features and highlighted that the hemihydrate maintains unaltered the antischistosomal activity and its physical state for three months at room temperature. Last but not least, both PZQ-HH aqueous solubility and intrinsic dissolution rate are largely superior to those of the commercially available Form A, counteracting the general rule that an anhydrous form is usually more soluble in water than the hydrated form.

A further important finding of this research is that the identity of PZQ crystal form used as starting material in the mechanochemical formation of PZQ-HH affects the course of the reaction. Single-step LAG of PZQ in water results in the hemihydrate when the reactant is PZQ anhydrous Form B, whereas PZQ-HH can only be obtained from anhydrous Form A via a two-step grinding mixed process using firstly, neat and subsequently, LAG conditions with water. The close relationship between PZQ-HH and anhydrous PZQ Form B can be traced to the similarity of the two crystal structures, which goes beyond the molecular conformation. Due to this similarity, Form B is more likely to support the nucleation of the hemihydrate phase, explaining the easier formation of the hemihydrate phase from this anhydrous polymorph. Similarly, in the case of dehydration of PZQ-HH, the similar packing arrangement of PZQ molecules in Form B explains the formation of this polymorph as a kinetically-preferred dehydration product, even though Form A would be the thermodynamically most favorable product.

## Figures and Tables

**Table 1:** Nature of Praziquantel polymorphs obtained by different processes.

Initial Polymorph	Method/Technique	Conditions/Duration	Outcome
A	LAG with water ^1^	Two-step ^2^	HH
A	LAG with water	One-step	A
B	LAG with water	One-step	HH
A	Slurry	7 days	A
B	Slurry	3 days ^3^	HH
A	LAG with water	One-step	HH
	with seeds of PZQ-HH		
HH	RT	4 months	B
HH	50 °C/under vacuum	Overnight	B
HH	Milling (25 Hz)	60 min	B
HH	25 °C–60 °C	Dynamic heating	B

^1^ The amount of water does not affect the final solid form. ^2^ In neat conditions for 30 min at 25 Hz, then, after addition of water, 1 h at 25 Hz. ^3^ Slurry experiments were conducted over seven days, periodically checking the solid; starting from day three the outcome was PZQ-HH.

**Table 2:** Experimental and calculated chemical shift for ^13^C SS-NMR analyses of PZQ-HH, compared to Form A, Form B and Form C, from refs. [19,20]. Atoms numbering refers to Figure 1.

Atom	Group	Form A	Form B	Form C	PZQ-HH	
		Exp	Exp	Exp	Exp	Calc ^1^
7′	C=O	175.4, 176.2 sh	173.6	173.3	173.6	173.8
4	C=O	165.8, 164.6, 162.1	164.3	163.4	165.4	165.4
7a	C_q_	137.7, 136.5	134.6	135.1	134.1	136.3
11a	C_q_	135.8, 134.6	134.6	134.0	134.1	136.2
8	CH	129.7, 127.5, 124.8	129.8	129.1	127.3	129.4
11	CH	133.7, 132.0, 130.5	127.5	127.5	127.3	129.9
10	CH		126.9	127.1	127.3	128.4
9	CH		125.8	127.1	124.0	124.7
11b	CH	56.3, 55.5	55.5	55.5	54.9	55.1
3	CH_2_	46.1	49.8	50.1	48.2	46.7
1	CH_2_	47.9	45.0	45.5	43.9	41.4
1′	CH	39.7	41.2	41.8	38.4	36.8
6	CH_2_	38.1	38.3	37.7	38.4	35.8
6’	CH_2_	32.0, 30.1, 27.9, 26.3, 25.3	29.4	27.4	25.8	24.5
2’	CH_2_		29.4	29.6	25.8	24.0
7	CH_2_		28.4	29.1	28.2	26.2
4’	CH_2_		26.7	25.4	25.8	23.5
3’	CH_2_		26.1	26.8	25.8	24.0
5’	CH_2_		25.0	25.4	27.3	25.3

^1^ see Section 2.2.12 Modelling of Solid-state NMR spectra.

## Supplementary Materials

The following are available online at https://www.mdpi.com/1999-4923/12/3/289/s1. Figure S1. PXRD pattern of (a) PZQ Form A and (b) sample obtained after 30 min of neat grinding (25 Hz). Figure S2. PXRD pattern of (a) PZQ-HH after compression in a hydraulic press and (b) fresh PZQ-HH. Figure S3. LAG experiments (30 min, 25 Hz) on Form A (raw PZQ) with different amounts of deionized water: with 10, 40, 60 and 100 μL of water. Figure S4. PXRD patterns of the solid residues after slurry in water of (a) PZQ Form A for 7 days and (b) PZQ Form B for 3 days. Figure S5. Hot-stage microscopy images for PZQ-HH. Figure S6. Comparison of experimental and calculated ^13^C SS-NMR spectrum of PZQ-HH. Figure S7. In situ PXRD of PZQ-HH upon heating from ambient temperature (top) to 80 °C (penultimate pattern). The progressive transition from PZQ-HH to Form B is visible. Further, Form B is stable at 80 °C: the pattern recorded at 25 °C (bottom) is superimposable to that of Form B. Table S1. Crystallographic parameters of the PZQ-HH structure determined from PXRD data. CCDC 1530464 contains the supplementary crystallographic data for racemic praziquantel hemihydrate (PZQ-HH) (These data can be obtained free of charge from The Cambridge Crystallographic Data Centre via www.ccdc.cam.ac.uk/structures).
