# Supplementary material for: Mechanochemical Formation of Racemic Praziquantel Hemihydrate with Improved Biopharmaceutical Properties
Source: Pharmaceutics. 2020 Mar 23;12(3):289. doi: 10.3390/pharmaceutics12030289 (PMC7151222; doi:10.3390/pharmaceutics12030289)
Supplement: Supplementary file 1 [file pharmaceutics-12-00289-s001.zip › pharmaceutics-750762-sup-for final/CCDC1530464- crystallographic information files/1530464_PZQ_hydr-data_PZQ_hydr_file002.html]

checkCIF/PLATON report


```
No syntax errors found.                               CIF dictionary  
Please wait while processing ....                     Interpreting this report
```

**Datablock: PZQ\_hydr**


---

|  |  |  |
| --- | --- | --- |
| Bond precision: | C-C = 0.0044 A | Wavelength=1.54060 |

|  |  |  |  |
| --- | --- | --- | --- |
| Cell: | a=5.85619(18) | b=10.9209(3) | c=14.2982(7) |
|  | alpha=105.753(3) | beta=94.628(3) | gamma=99.553(2) |
| Temperature: | 293 K |  |  |

|  |  |  |
| --- | --- | --- |
|  | Calculated | Reported |
| Volume | 860.24(6) | 860.24(6) |
| Space group | P -1 | P -1 |
| Hall group | -P 1 | -P 1 |
| Moiety formula | 2(C19 H24 N2 O2), H2 O | 2(C19 H24 N2 O2), H2 O |
| Sum formula | C38 H50 N4 O5 | C38 H50 N4 O5 |
| Mr | 642.82 | 642.82 |
| Dx,g cm-3 | 1.241 | 1.241 |
| Z | 1 | 1 |
| Mu (mm-1) | 0.630 | 0.000 |
| F000 | 346.0 | 346.0 |
| F000' | 346.99 |  |
| h,k,lmax | 3,7,9 |  |
| Nref | 491 |  |
| Tmin,Tmax |  |  |
| Tmin' |  |  |

|  |  |
| --- | --- |
| Correction method= Not given |  |

|  |  |
| --- | --- |
| Data completeness= 0.000 | Theta(max)= |

|  |  |
| --- | --- |
| R(reflections)= | wR2(reflections)= |

S = | Npar= |

---

```
The following ALERTS were generated. Each ALERT has the format
       test-name_ALERT_alert-type_alert-level.
Click on the hyperlinks for more details of the test.


---
```

---

---

It is advisable to attempt to resolve as many as possible of the alerts in all categories. Often the minor alerts point to easily fixed oversights, errors and omissions in your CIF or refinement strategy, so attention to these fine details can be worthwhile. In order to resolve some of the more serious problems it may be necessary to carry out additional measurements or structure refinements. However, the purpose of your study may justify the reported deviations and the more serious of these should normally be commented upon in the discussion or experimental section of a paper or in the "special\_details" fields of the CIF. checkCIF was carefully designed to identify outliers and unusual parameters, but every test has its limitations and alerts that are not important in a particular case may appear. Conversely, the absence of alerts does not guarantee there are no aspects of the results needing attention. It is up to the individual to critically assess their own results and, if necessary, seek expert advice. **Publication of your CIF in IUCr journals** A basic structural check has been run on your CIF. These basic checks will be run on all CIFs submitted for publication in IUCr journals (*Acta Crystallographica*, *Journal of Applied Crystallography*, *Journal of Synchrotron Radiation*); however, if you intend to submit to *Acta Crystallographica Section C* or *E* or *IUCrData*, you should make sure that full publication checks are run on the final version of your CIF prior to submission. **Publication of your CIF in other journals** Please refer to the *Notes for Authors* of the relevant journal for any special instructions relating to CIF submission. |

---

**PLATON version of 24/11/2016; check.def file version of 23/11/2016**

|  |
| --- |
| **Datablock PZQ\_hydr** - ellipsoid plot |
|  |

---

 Download CIF editor (publCIF) from the IUCr   
 Download CIF editor (enCIFer) from the CCDC   
 Test a new CIF entry 
