# Supplementary material for: Mechanochemical Formation of Racemic Praziquantel Hemihydrate with Improved Biopharmaceutical Properties
Source: Pharmaceutics. 2020 Mar 23;12(3):289. doi: 10.3390/pharmaceutics12030289 (PMC7151222; doi:10.3390/pharmaceutics12030289)
Supplement: Supplementary file 1 [file pharmaceutics-12-00289-s001.zip › pharmaceutics-750762-sup-for final/CCDC1530464- crystallographic information files/1530464_PZQ_hydr-data_PZQ_hydr_file002.pdf]

No syntax errors found.
Please wait while processing ....

CIF dictionary
Interpreting this report

Datablock: PZQ_hydr

Bond precision:	C-C = 0.0044 A		Wavelength=1.54060
Cell:	a=5.85619(18)	b=10.9209(3)	c=14.2982(7)
	alpha=105.753(3)	beta=94.628(3)	gamma=99.553(2)
Temperature :	293 K		

	Calculated	Reported
Volume	860.24(6)	860.24(6)
Space group	P -1	P -1
Hall group	-P 1	-P 1
Moiety formula	2(C19 H24 N2 O2), H2 O	2(C19 H24 N2 O2), H2 O
Sum formula	C38 H50 N4 O5	C38 H50 N4 O5
Mr	642.82	642.82
Dx,g cm-3	1.241	1.241
Z	1	1
Mu (mm-1)	0.630	0.000
F000	346.0	346.0
F000'	346.99	
h,k,lmax	3,7,9	
Nref	491	
Tmin,Tmax		
Tmin'		

Correction method= Not given

Data completeness= 0.000 Theta(max)=

R(reflections)= wR2(reflections)=

S = Npar=

The following ALERTS were generated. Each ALERT has the format
test-name_ALERT_alert-type_alert-level.
Click on the hyperlinks for more details of the test.

Alert level C

REFI015_ALERT_1_C _refine_ls_shift/su_max is missing
Maximum shift/s.u. ratio after final refinement cycle.
The following tests will not be performed
SHFSU_01

PLAT340_ALERT_3_C Low Bond Precision on C-C Bonds .... 0.00444 Ang.
PLAT410_ALERT_2_C Short Intra H...H Contact H1 .. H14 .. 1.98 Ang.
PLAT790_ALERT_4_C Centre of Gravity not Within Unit Cell: Resd. # 1 Note
C19 H24 N2 O2

Alert level G

PLAT092_ALERT_4_G Check: Wavelength given is not Cu,Ga,Mo,Ag,In Ka 1.54060 Ang.
PLAT300_ALERT_4_G Atom Site Occupancy of *O3 is Constrained at 0.5 Check
And 2 other PLAT300 Alerts

PLAT300_ALERT_4_G Atom Site Occupancy of *H25 is Constrained at 0.5 Check
PLAT300_ALERT_4_G Atom Site Occupancy of *H26 is Constrained at 0.5 Check

PLAT302_ALERT_4_G Anion/Solvent/Minor-Residue Disorder (Resd 2).. 100 % Note
PLAT304_ALERT_4_G Non-Integer Number of Atoms (1.50) in Resd. # 2 Check
PLAT432_ALERT_2_G Short Inter X...Y Contact O3 .. C2 .. 2.83 Ang.
PLAT793_ALERT_4_G The Model has Chirality at C3 (Centro SPGR) R Verify
PLAT981_ALERT_1_G No non-zero f" Anomalous Scattering Values Found Please Check
PLAT986_ALERT_1_G No non-zero f' Anomalous Scattering Values Found Please Check

0 **ALERT level A** = Most likely a serious problem - resolve or explain
0 **ALERT level B** = A potentially serious problem, consider carefully
4 **ALERT level C** = Check. Ensure it is not caused by an omission or oversight
10 **ALERT level G** = General information/check it is not something unexpected

```
3 ALERT type 1 CIF construction/syntax error, inconsistent or missing data
2 ALERT type 2 Indicator that the structure model may be wrong or deficient
1 ALERT type 3 Indicator that the structure quality may be low
8 ALERT type 4 Improvement, methodology, query or suggestion
0 ALERT type 5 Informative message, check
```

It is advisable to attempt to resolve as many as possible of the alerts in all categories. Often the minor alerts point to easily fixed oversights, errors and omissions in your CIF or refinement strategy, so attention to these fine details can be worthwhile. In order to resolve some of the more serious problems it may be necessary to carry out additional measurements or structure refinements. However, the purpose of your study may justify the reported deviations and the more serious of these should normally be commented upon in the discussion or experimental section of a paper or in the "special_details" fields of the CIF. checkCIF was carefully designed to identify outliers and unusual parameters, but every test has its limitations and alerts that are not important in a particular case may appear. Conversely, the absence of alerts does not guarantee there are no aspects of the results needing attention. It is up to the individual to critically assess their own results and, if necessary, seek expert advice.

Publication of your CIF in IUCr journals

A basic structural check has been run on your CIF. These basic checks will be run on all CIFs submitted for publication in IUCr journals (*Acta Crystallographica*, *Journal of Applied Crystallography*, *Journal of Synchrotron Radiation*); however, if you intend to submit to *Acta Crystallographica Section C* or *E* or *IUCrData*, you should make sure that full publication checks are run on the final version of your CIF prior to submission.

Publication of your CIF in other journals

Please refer to the *Notes for Authors* of the relevant journal for any special instructions relating to CIF submission.

PLATON version of 24/11/2016; check.def file version of 23/11/2016

Datablock PZQ_hydr - ellipsoid plot

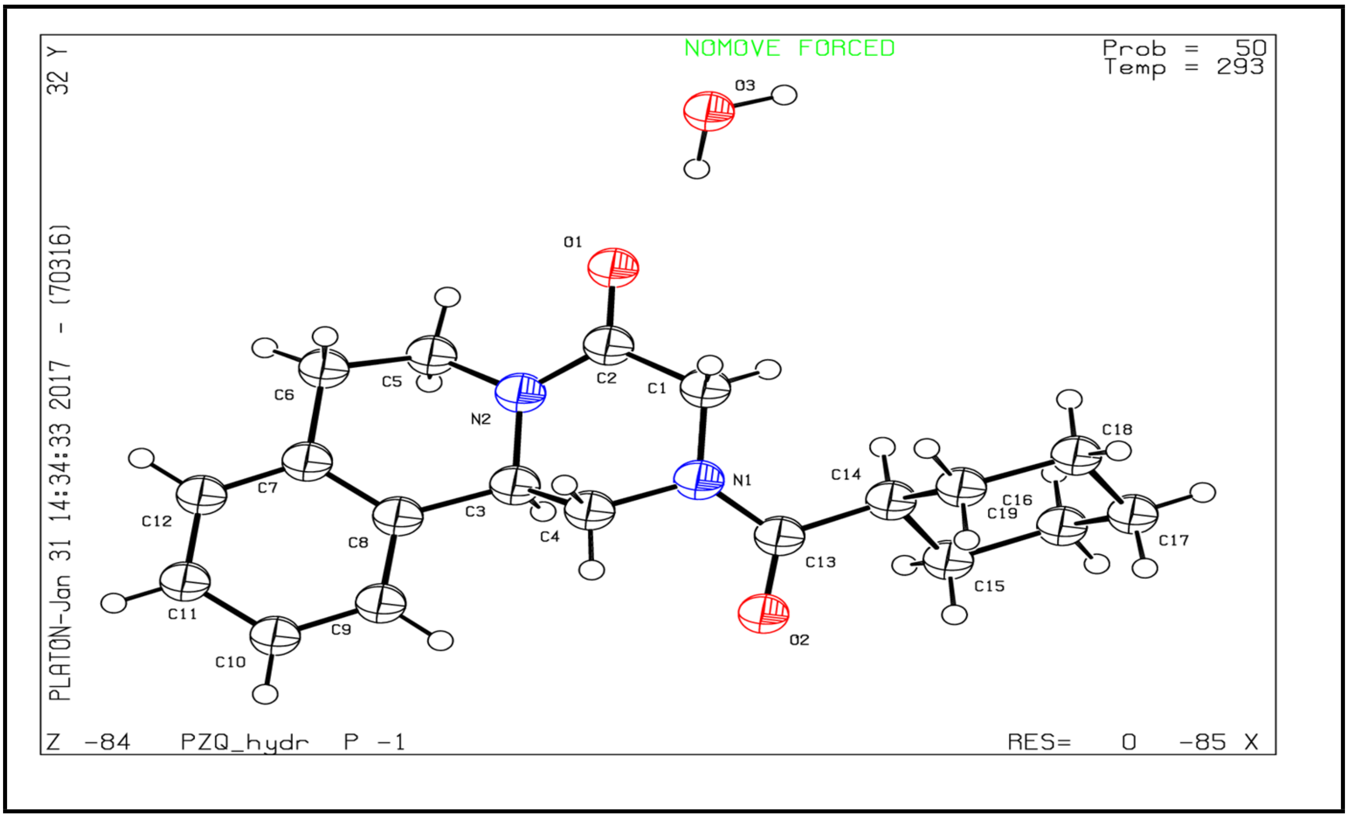

Download CIF editor (publCIF) from the IUCr
Download CIF editor (enCIFer) from the CCDC
Test a new CIF entry
