# Supplementary material for: Mechanochemical Formation of Racemic Praziquantel Hemihydrate with Improved Biopharmaceutical Properties
Source: Pharmaceutics. 2020 Mar 23;12(3):289. doi: 10.3390/pharmaceutics12030289 (PMC7151222; doi:10.3390/pharmaceutics12030289)
Supplement: Supplementary file 1 [file pharmaceutics-12-00289-s001.zip › pharmaceutics-750762-sup-for final/pharmaceutics-750762-supplementary.docx]

Supplementary Materials: Mechanochemical Formation of Racemic Praziquantel Hemihydrate with Improved Biopharmaceutical Properties

Debora Zanolla, Dritan Hasa, Mihails Arhangelskis, Gabriela Schneider-Rauber,
Michele R. Chierotti, Jennifer Keiser, Dario Voinovich, William Jones and Beatrice Perissutti

**
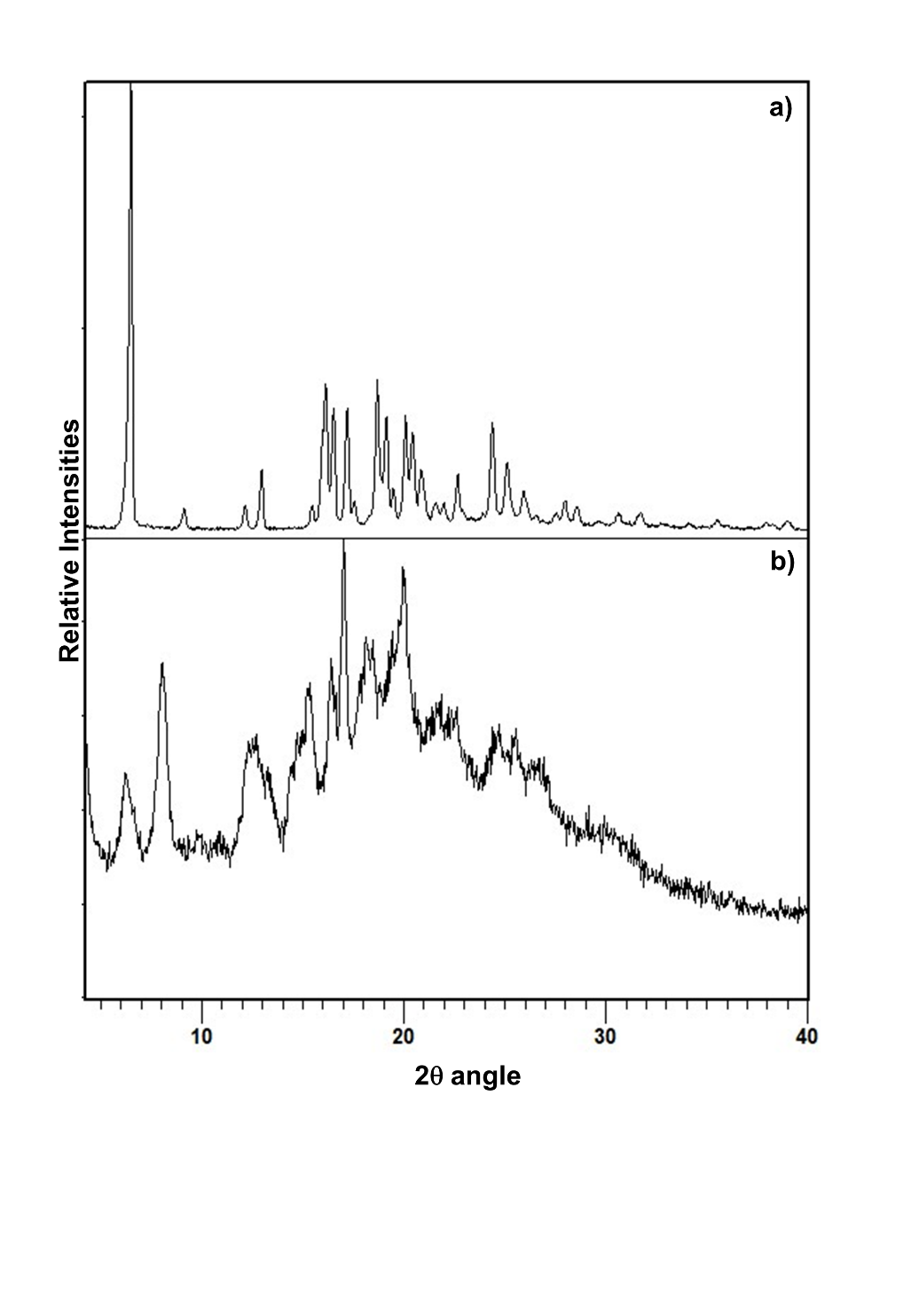
**

**Figure S1.** PXRD pattern of (**a**) PZQ Form A and (**b**) sample obtained after 30 min of neat grinding (25 Hz).


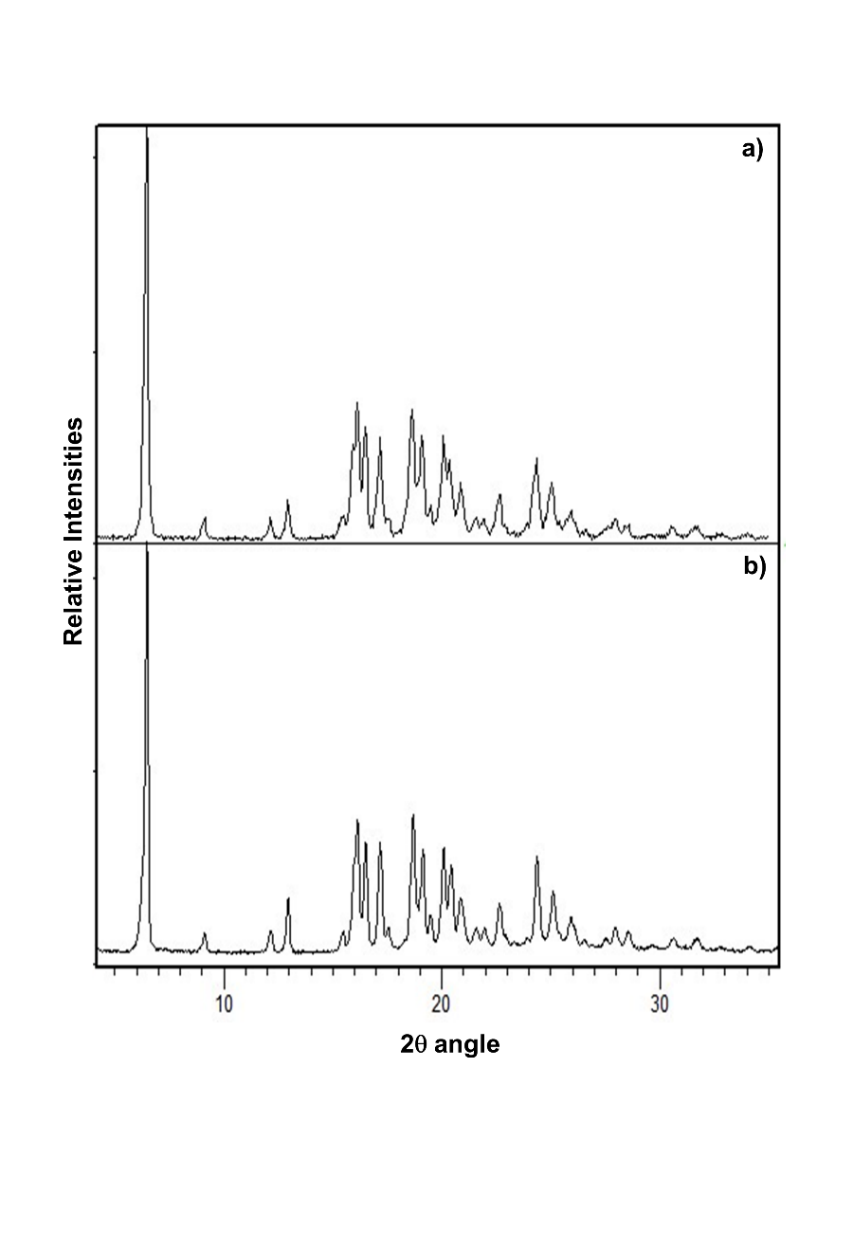


**Figure S2.** PXRD pattern of (**a**) PZQ-HH after compression in a hydraulic press and (**b**) fresh PZQ-HH.

**Figure S3.** LAG experiments (30 min, 25 Hz) on Form A (raw PZQ) with different amounts of deionized water: with 10, 40, 60 and 100 μL of water.


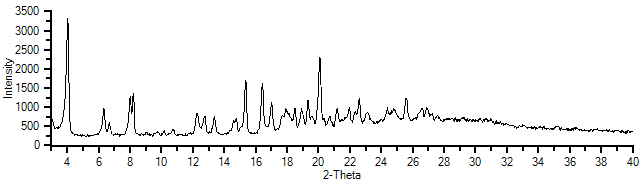

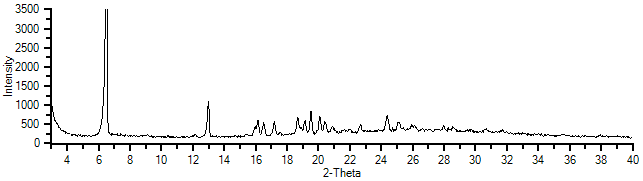


a)

b)

**Figure S4.** PXRD patterns of the solid residues after slurry in water of (**a**) PZQ Form A for 7 days and (**b**) PZQ Form B for 3 days.

**Figure S5.** Hot-stage microscopy images for PZQ-HH.

**Figure S6.** Comparison of experimental and calculated ^13^C SS-NMR spectrum of PZQ-HH.


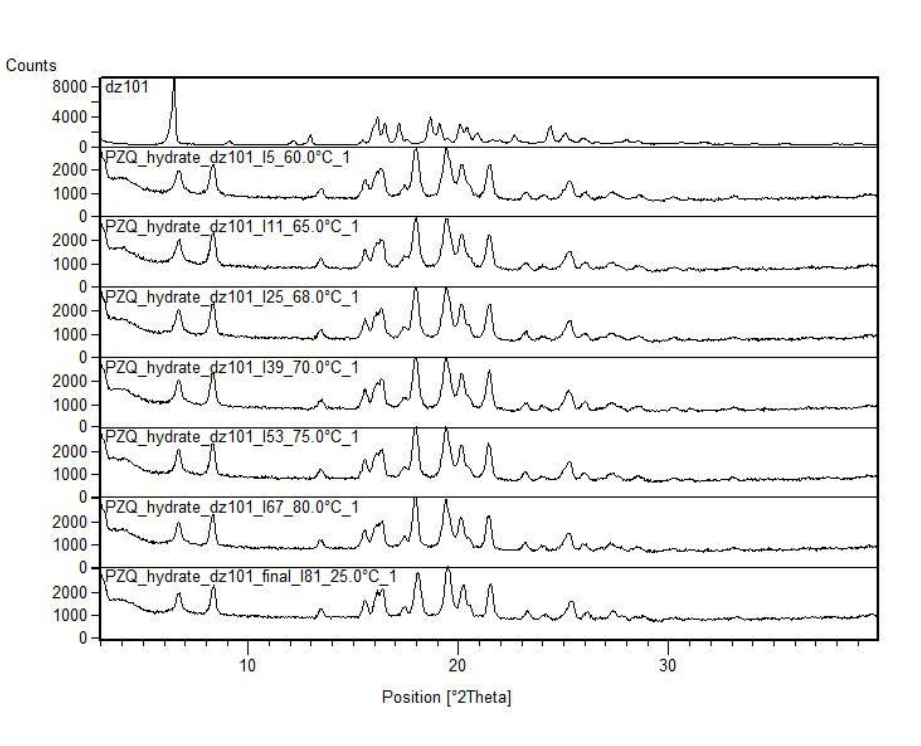


**Figure S7.** In situ PXRD of PZQ-HH upon heating from ambient temperature (top) to 80 °C (penultimate pattern). The progressive transition from PZQ-HH to Form B is visible. Further, Form B is stable at 80 °C: the pattern recorded at 25 °C (bottom) is superimposable to that of Form B.

**Table S1.** Crystallographic parameters of the PZQ-HH structure determined from PXRD data.

| Compound | Praziquantel hemihydrate |
| --- | --- |
| Chemical formula | (C_19_H_24_N_2_O_2_)_2_ H_2_O |
| *M*_r_/g mol^-1^ | 642.82 |
| Crystal system | triclinic |
| Space group | *P-1* |
| F(000) | 346 |
| X-ray radiation type | CuK_α1_ |
| *a/Å* | 5.8562(2) |
| *b/Å* | 10.9209(3) |
| *c/Å* | 14.2982(7) |
| *α/º* | 105.753(3) |
| *β/º* | 94.628(3) |
| *γ/º* | 99.553(3) |
| *V/Å^3^* | 860.24(6) |
| Z | 1 |
| *ρ*_calc_/g cm^–3^ | 1.2409(1) |
| *R_p_*/% | 4.3 |
| *R_wp_*/% | 5.3 |
| R_Bragg_ | 1.6 |
| *χ2* | 1.646 |
| CCDC number | 1530464 |
